# Endothelial dysfunction in a mouse model of human neutral lipid storage disease

**DOI:** 10.1186/2050-6511-14-S1-P64

**Published:** 2013-08-29

**Authors:** Astrid Schrammel, Marion Mussbacher, Gerald Wölkart, Heike Stessel, Günter Hämmerle, Wael Al Zoughbi, Gerald Höfler, Alois Lametschwandtner, Rudolf Zechner, Bernd Mayer

**Affiliations:** 1 Department of Pharmacology and Toxicology, Institute of Pharmaceutical Sciences, University of Graz, 8010 Graz, Austria; 2 Department of Molecular Bioscience, University of Graz, 8010 Graz, Austria; 3 Institute of Pathology, Medical University of Graz, Auenbruggerplatz 25, 8010 Graz, Austria; 4 Department of Cell Biology and Physiology, Vessel and Muscle Research Unit, University of Salzburg, 5020 Salzburg, Austria

## Background

Systemic knockout of adipose triglyceride lipase (ATGL), the rate-limiting enzyme of triglyceride catabolism, results in a murine phenotype characterized by progressive accumulation of lipids in the heart finally leading to lethal cardiac dysfunction. Since cardiac and vascular dysfunction are closely related we investigated endothelium-dependent and -independent vessel function of ATGL knockout (ATGL(-/-)) mice. Using mice with cardiomyocyte-restricted overexpression of ATGL (cardiac-rescued phenotype;

ATGL(-/-)/MHC-A35) we were able to differentiate between heart-related and -unrelated effects.

## Results

Aortic relaxation studies and Langendorff perfusion experiments of isolated hearts demonstrated that ATGL(-/-) mice suffer from pronounced micro- and macrovascular endothelial dysfunction. Experiments with DEA/NO revealed the functional integrity of the smooth muscle cell layer. Since loss in vascular reactivity was restored by ~50 % in cardiac-rescued mice, this phenomenon seems partly a consequence of impaired cardiac contractility. Biochemical analysis revealed that aortic eNOS protein was down-regulated by more than 60% in aortas of ATGL(-/-) mice. As consequence, phosphorylation of VASP at Ser 239 was almost abolished. Both parameters were totally reversed in vessels of cardiac rescued mice. Aortic expression of eNOS mRNA was not affect in ATGL deficiency excluding a transcriptional mechanism underlying the observed effect. Total levels of ubiquitinated proteins (a measure of vascular proteasomal activity) were down-regulated in ATGL-deficient aortas (~26% compared to WT controls) and fully recovered upon cardiomyocyte-restricted overexpression of ATGL.

**Figure 1:**
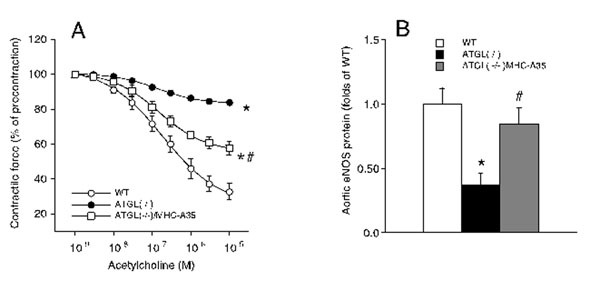
**(A)** Aortic relaxation to acetylcholine is blunted in ATGL deficiency and partly recovered in vessels of mice with a cardiac rescued phenotype. **(B)** Aortic eNOS expression in downregulated in ATGL deficiency an reversed upon cardiac rescue.

## Conclusion

Endothelial dysfunction in murine ATGL deficiency partly arises from impaired cardiac contractility and originates from down-regulation of aortic eNOS presumably due to activation of the vascular proteasome. Potential heart-independent mechanisms contributing to the observed defect are currently investigated.

